# Myeloid-Derived Suppressor Cells Alleviate Renal Fibrosis Progression *via* Regulation of CCL5-CCR5 Axis

**DOI:** 10.3389/fimmu.2021.698894

**Published:** 2021-09-10

**Authors:** Yue Qiu, Yirui Cao, Guowei Tu, Jiawei Li, Ying Su, Fang Fang, Xuepeng Zhang, Jing Cang, Ruiming Rong, Zhe Luo

**Affiliations:** ^1^ Department of Critical Care Medicine, Zhongshan Hospital, Fudan University, Shanghai, China; ^2^ Department of Anesthesiology, Zhongshan Hospital, Fudan University, Shanghai, China; ^3^ Department of Urology, Zhongshan Hospital, Fudan University, Shanghai, China; ^4^ Shanghai Key Laboratory of Organ Transplantation, Shanghai, China; ^5^ Department of Critical Care Medicine, Xiamen Branch, Zhongshan Hospital, Fudan University, Xiamen, China

**Keywords:** renal fibrosis, CCL5, CCR5, migration, myeloid-derived suppressor cells

## Abstract

**Background:**

Renal fibrosis is inevitable in all progressive chronic kidney diseases (CKDs) and represents a serious public health problem. Immune factors contribute to the progression of renal fibrosis. Thus, it is very possible that immunosuppression cells, such as myeloid-derived suppressor cells (MDSCs), could bring benefits to renal fibrosis. Herein, this study investigated the antifibrotic and reno-protective effect of MDSCs and the possible mechanisms.

**Methods:**

Murine and cell models of unilateral ureter obstruction (UUO) renal fibrosis were used. Bone marrow-induced MDSCs and granulocyte–macrophage colony-stimulating factor (GM-CSF) were pretreated before surgery. Kidney weight, pathological injury, extracellular matrix deposition, and epithelial–mesenchymal transition progression were examined. Transforming growth factor (TGF)-β1)/Smad/Snail signaling pathway involvement was investigated through Western blotting and quantitative PCR (qPCR). Accumulation of MDSC, CD4+ T cell, regulatory T (Treg), and T helper 1 (T_H_1) cell accumulation, and CCL5 and CCR5 expression level in MDSCs and non-MDSCs were evaluated using flow cytometry.

**Results:**

*In vitro*- and *in vivo-*induced MDSCs significantly ameliorated UUO-induced tubulointerstitial fibrosis, inhibited the TGF-β1/Smad/Snail signaling pathway, and enhanced MDSC and Treg infiltration in the kidney while downregulating the T_H_1 cells. Both *in vitro* and *in vivo* experiments confirmed CCL5 elevation in the two MDSC-treated groups.

**Conclusion:**

*In vitro*- and *in vivo*-induced MDSCs alleviated renal fibrosis similarly through promoting the CCL5–CCR5 axis interaction and TGF-β1/Smad/Snail signaling pathway inhibition. Our results indicate an alternative treatment for renal fibrosis.

## Introduction

Renal fibrosis is the common outcome of all progressive chronic kidney diseases (CKDs), which brings a great burden to public health (1). Excessive extracellular matrix (ECM) deposition and epithelial–mesenchymal transition (EMT) play essential roles during disease deterioration. Moreover, ameliorating or reversing renal fibrosis progression is difficult. Hence, it is of great urgency to explore feasible treatments to address this challenge.

Several immune factors are reported to contribute to the progression of renal fibrosis (2–4), including immune cells, inflammatory cytokines, chemokine receptors, and their ligands (5–7). Targeted mainly on T cells, myeloid-derived suppressor cells (MDSCs) are a distinctive myeloid cells population with strong immunosuppressive capacity. In mice, MDSCs are characterized by CD11b (integrin α-M) and myeloid differentiation antigen Gr-1 expression (8). Adoptive transference of MDSCs have an excellent performance in autoimmune disease treatment and allograft protection (9–11). Recently, their treating effects have also been confirmed in acute renal injury and chronic renal fibrosis (12). However, the mechanism through which MDSCs ameliorate renal fibrosis remains unclear.

Granulocyte colony-stimulating factor (G-CSF) and granulocyte–macrophage colony stimulating factor (GM-CSF) are capable of inducing *in vitro* and *in vivo* MDSCs expansion (9, 13). Recent studies have indicated that G-CSF pretreatment attenuates acute ischemia–reperfusion injury and subsequent renal fibrosis by increasing MDSC infiltration into the kidney (12). GM-CSF also stimulates neutrophils to MDSCs conversion in early human pregnancy (14). Since there is also an evidence that bone marrow (BM)-induced MDSCs exhibit more potent suppressive function than mice-isolated MDSCs (13), here raises new questions: is there a difference in the function of MDSCs induced *in vitro* or *in vivo*? Is there another mechanism that mediates the enhanced infiltration of MDSCs into renal injury?

Interactions between chemokines and chemokine receptors are involved in MDSC recruitment (15), such as C–X–C motif chemokine ligand (CXCL) 8 and its receptor (16), CXCL1-CXCR2 (17), and C–C chemokine receptor (CCR) 5 and its ligands (18, 19). The C–C motif chemokine ligand (CCL) 5–CCR5 axis, a potential therapeutic target for cancer (19) and human immunodeficiency virus (HIV) (20) infection, is highly involved in MDSC-mediated immunosuppression (21). A previous study has showed that interleukin (IL)-6 and GM-CSF upregulate CCR5 expression during *in vitro* MDSC induction (18). However, in renal fibrosis, whether the CCL5–CCR5 axis plays a role in MDSC recruitment and if there is a difference in the expression level of this axis between *in vivo*- and *in vitro*-induced MDSCs remain unknown.

The transforming growth factor (TGF)-β1/Smad signaling pathway is one of the canonical fibrogenic growth signaling pathways and considered one of the most important renal fibrosis regulators (22–24). The Smad complex is formed and transferred to the nucleus to modulate the expression of target genes after TGF-β1-triggered Smad2 and Smad3 phosphorylation. Among these target genes, Snail is a ubiquitous zinc-finger transcription factor that suppresses E-cadherin expression and accelerates EMT (25) and fibrosis (26, 27). However, it is unclear whether the TGF-β1/Smad/Snail signaling pathway is involved in the renoprotective effect of MDSCs.

In this study, BM and GM-CSF were used to induce MDSCs *in vitro* and *in vivo*, respectively, and their efficacy on renal fibrosis progression was evaluated. To further elucidate the potential mechanisms, the TGF-β1/Smad/Snail signaling pathway and the interaction between CCL5–CCR5 axis were examined. Our results indicate an alternative method for renal protection during CKD.

## Materials and Methods

### Animals and Unilateral Ureter Obstruction-Induced Renal Fibrosis Model

Male 6- to 8-week-old C57BL/6J mice weighing 20–25 g were obtained from the Shanghai SLAC Laboratory Animal Co., Ltd., and bred in a specific pathogen-free (SPF)-grade animal room. All animal procedures were approved by the Animal Ethical Committee of Zhongshan Hospital, Fudan University. Mice were allocated randomly into four groups (n = 6): (1) sham group, (2) unilateral ureter obstruction (UUO) group, (3) UUO + MDSC group (MDSC), and (4) UUO + GM-CSF group (GM-CSF). The UUO-induced renal fibrosis model was established as previously described (28). Briefly, the mice were anesthetized with 100 mg/kg pentobarbital intraperitoneally. After the midline incision, the left kidney was exposed. The left ureter was isolated and ligated at two points with non-absorbable 4–0 silk sutures and then cut between the ligatures. Sterile saline (1 mL) was administered intraperitoneally before the abdominal closure. In the sham group, the left ureter was isolated but not ligated. In the MDSC group, mice were pretreated with 1 × 10^5^ BM-induced MDSCs *via* the tail vein just before the surgery. In the GM-CSF group, 10 µg GM-CSF (PeproTech; RH, USA) was administered intraperitoneally for seven consecutive days prior to UUO. The animals were sacrificed 10 days after the operation.

### Cell Culture and Treatment

Mouse renal tubular epithelial cells (mTECs) were purchased from the American Type Culture Collection (ATCC) and cultured in Dulbecco’s modified Eagle’s medium (DMEM) supplemented with 10% fetal bovine serum (FBS) at 37°C and 5% CO_2_ for 24 h. They were divided into four groups for different treatments and seeded into 24-well plates (1 × 10^5^ cells/well): (1) control, (2) TGF-β1, (3) TGF-β1 + 1 × 10^4^ MDSCs (MDSC-L), and (4) TGF-β1 + 1 × 10^5^ MDSCs (MDSC-H). Each group was administered with 10 ng/ml TGF-β1, except the control, which was administered with the same amount of sterile phosphate-buffered saline (PBS). All cells were further cultured for 24 h before being harvested and processed for Western blot and flow cytometry analyses.

### *In Vitro* Induction, Isolation, and Adoptive Transference of MDSC

BM cells collected from 6- to 8-week-old C57BL/6 mice tibia and femur were flushed with PBS. Red blood cell (RBC) lysis buffer (BD Biosciences; CA, USA) was used to lyse red blood cells. Cells (2 × 10^6^) were cultured at 37°C and 5% CO_2_ for 7 days in 6 cm dishes containing 3 ml Roswell Park Memorial Institute (RPMI)-1640 with 10% FBS, 1% streptomycin and penicillin, 1% MEM nonessential amino acids (NEAA) solution, 1% sodium pyruvate (Gibco, NY, USA), 2 μl 2-mercaptoethanol (Sigma-Aldrich, St. Louis, USA), 50 ng/ml GM-CSF (PeproTech), and 40 ng/ml interleukin (IL)-6 (PeproTech). The cells were harvested for testing or sorting by FACS Aria III (BD Biosciences, CA, USA) using CD11b-fluorescein isothiocyanate (FITC) (eBioscience, CA, USA) and Gr-1-PerCP Cy5.5 (eBioscience). Flow cytometry verified that all isolated MDSCs yielded >90% pure population. The purified MDSCs were then adoptively transferred to mice *via* the tail vein just before UUO. For cell tracing, some UUO mice were injected with MDSCs and stained with CFSE (Invitrogen).

### Single-Cell Suspension Preparation

One-fourth kidney from the damaged side was dissected and finely minced with a gentleMACS Dissociator (Miltenyi Biotec, CA, USA) in Hank’s buffer without Ca^2+^ (Gibco) containing 10% type IV collagenase (Gibco) and 0.002% DNase I (Invitrogen). The spleen was ground and filtered through a 70-μm cell mesh and resuspended in PBS. BM cells were flushed with PBS. Red blood cells were lysed for all the single-cell suspensions. The cells were then filtered and resuspended in PBS + 10% FBS for further experiments.

### Cell Staining and Flow Cytometry

The cells were divided into several groups and then stained with fluorophore-conjugated antibodies, including CD11b-FITC or BV421 (eBioscience), Gr-1-PerCP Cy5.5 or APC (eBioscience), CCR5-APC or PE (BioLegend, CA, USA), CD45-AmCyan (BioLegend), CD4-PE (eBioscience), and CD25-FITC (eBioscience) for 30 min at 4°C in staining buffer (PBS with 10% FBS). CCL5-PE/Cyanine7 (BioLegend), Foxp3-APC (eBioscience), and interferon-gamma (IFN-γ)-FITC (eBioscience) were used for intracellular staining after culturing with fixation/permeabilization medium (eBioscience). Data were acquired through FACS AriaIII (BD Biosciences) and analyzed with FlowJo X (BD Biosciences).

### Pathological Assessment

Paraffin-embedded one-fourth injured kidney was cut into 5-mm sections from the maximum side and subsequently deparaffinized and rehydrated. Hematoxylin and eosin (H&E) staining, Masson trichrome, and Sirius red staining were performed as previously described. Ten fields of each specimen were randomly chosen for observation at 200× magnification. For H&E staining, the severity of histological injury was evaluated randomly and independently by two renal pathologists according to the grading system described by Lin et al. Semiquantitative assessment of Sirius red and Masson trichrome was performed using ImageJ bundled with Java 1.8.0_172 (Media Cybernetics, MD, USA) to evaluate ECM deposition in the renal interstitium.

### Immunofluorescence Staining

α-Smooth muscle actin (SMA) and E-cadherin were detected after staining using a rabbit anti-SMA primary antibody (Abcam, Cambridge, UK) with Alexa Fluor 594-donkey antirabbit secondary antibody (1:1,000; Invitrogen, MA, USA) and a mouse anti-E-cadherin primary antibody (1:200; Cell Signaling Technology, Boston, MA, USA) with Alexa Fluor 488-donkey antimouse antibody (1:200, Invitrogen). Finally, 4′,6-diamidino-2-phenylindole (DAPI) was used for counter-staining.

### Western Blot Analysis

Western blot analysis was performed as described previously (29). Briefly, the total protein was extracted from kidney tissue samples or cultured cells (15 μg each) and separated using electrophoresis. After transferring on a polyvinylidene difluoride (PVDF) membrane, the membrane was incubated with 1:1,000 diluted primary antibodies, including anti-α-SMA, anticollagen-I, antifibronectin (all from Abcam), anti-E-cadherin, anti-p-Smad3, anti- Smad2/3, glyceraldehyde 3-phosphate dehydrogenase (GAPDH) (all from Cell Signaling Technology), anti-p-Smad2, anti-Snail (all from Beyotime; SH, China), and anti-CCL5 (ABclonal; WH, China). Goat antirabbit secondary antibody (Abcam) was added and visualized using an enhanced chemiluminescence system (Thermo Fisher Scientific, IL, USA). Band intensity was analyzed using ImageJ software and normalized to the value of GAPDH.

### Quantitative Real-Time PCR

Total RNA was extracted from kidney tissue using TRIzol reagent (Invitrogen, Carlsbad, CA, USA). Then, 1 µg RNA was reverse transcribed into complementary DNA (cDNA) using HiScript II Q RT SuperMix for qPCR (Vazyme, NJ, China) in accordance with the manufacturer’s instructions. Real-time PCR was performed as described in our previous report using a Mastercycle reprealplex system (Eppendorf, Hamburg, Germany). Target gene expression levels were evaluated by reference to the value of GAPDH using the 2^−ΔΔCt^ method. The PCR primers (including those for fibronectin, collagen I, E-cadherin, α-SMA, TGF-β1, Snail, and GAPDH) were synthesized according to sequences provided by PrimerBank (**Table 1**).

**Table 1:** Primers sequences used for RT-PCR.

Gene	Primers
Fibronectin	F: 5′-TGGAGAGACAGGAGGAAATAGC-3′
	R: 5′-CAGTGACAGCATACAGGGTGAT-3′
Collagen I	F: 5′-GAGAGAGCATGACCGATGGA-3′
	R: 5′-CGTGCTGTAGGTGAATCGAC-3′
E-cadherin	F: 5′-CAGGTCTCCTCATGGCTTTGC-3′
	R: 5′-CTTCCGAAAAGAAGGCTGTCC-3′
α-SMA	F: 5′-GTCCCAGACATCAGGGAGTAA-3′
	R: 5′-TCGGATACTTCAGCGTCAGGA-3′
TGF-β1	F: 5′-CTCCCGTGGCTTCTAGTGC-3′
	R: 5′-GCCTTAGTTTGGACAGGATCTG-3′
Snail	F: 5′-CACACGCTGCCTTGTGTCT-3′
	R: 5′-GGTCAGCAAAAGCACGGTT-3′
GAPDH	F: 5′-TCACCATCTTCCAGGAGCGAGAC-3′
	R:5′-TGAGCCCTTCCACAATGCCAAAG-3′

### Enzyme-Linked Immunosorbent Assay for Serum TGF-β1

TGF-β1 levels in the serum samples were measured using a commercially available ELISA kit (MLBio, Shanghai, China) according to the manufacturer’s instructions.

### Statistical Analysis

Data are presented as means ± standard deviation (SD). GraphPad Prism 9 (GraphPad Software Inc., CA, USA) was used for the statistical analyses. Two-tailed independent Student’s *t*-test was applied for comparison between two groups and one-way analysis of variance (ANOVA) followed by Bonferroni’s correction for more groups. Statistical significance was set at *p* < 0.05.

## Results

### BM-Induced MDSCs and GM-CSF Alleviate Renal Fibrosis

**Figure 1:**
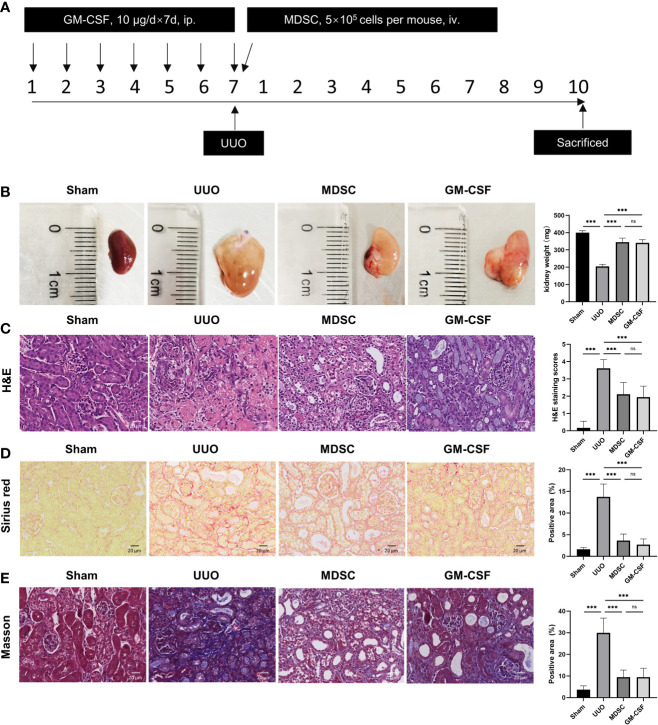
BM-induced MDSCs and GM-CSF alleviate renal fibrosis induced by UUO. **(A)** Flow chart of the *in vivo* study. **(B)** Kidney size and weight at 10 days after UUO. **(C)** Representative micrographs of H&E staining of kidney sections (scale bar, 20 μm) and semiquantitative data of tubule injury score. **(D)** Representative micrographs of Sirius red staining and semiquantitative data of fibrosis area. **(E)** Representative micrographs of Masson staining and semiquantitative data of fibrosis area. Data are presented as the mean ± SD (n = 6). ***p < 0.001. ns^P > 0.05.

Mice kidney were harvested 10 days after surgery (**Figure 1A**). The kidney weight significantly decreased on the ligation side, and the pelvis and calices were severely dilated with atrophied renal parenchyma in the UUO group (**Figure 1B**). H&E staining showed severe tubular dilation, interstitial expansion, and inflammatory cell infiltration (**Figure 1C**). However, kidney weight increased in the MDSC or GM-CSF groups (**Figure 1B**), and renal fibrosis injury was significantly alleviated (**Figure 1C**). There were no significant differences between the two groups.

Collagen deposition was detected using Sirius red and Masson staining (**Figures 1D, E**, respectively). Compared with that in the sham group, collagen accumulation in the renal interstitium increased significantly in UUO groups, while MDSC transplantation and GM-CSF pretreatment decreased interstitial collagen deposition, although these results were not comparable with those of the sham group. This evidence indicates a definite and similar renoprotective effect of MDSC transplantation and GM-CSF pretreatment.

### BM-Induced MDSCs and GM-CSF Reduce ECM Deposition and EMT

**Figure 2:**
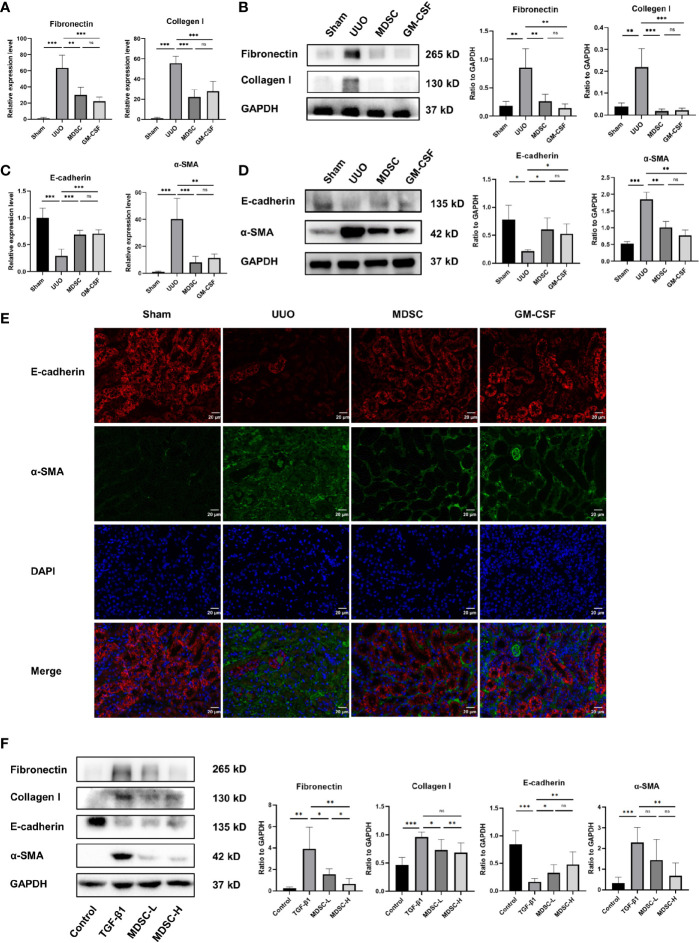
BM-induced MDSCs and GM-CSF reduce ECM deposition and EMT. **(A)** Relative mRNA levels of fibronectin and collagen I in renal tissue. **(B)** Western blot analysis of fibronectin and collagen I in renal tissue and their relative expression level to GAPDH. **(C)** Relative mRNA levels of E-cadherin and α-SMA in renal tissue. **(D)** Western blot analysis of E-cadherin and α-SMA in renal tissue and their relative expression level to GAPDH. **(E)** Representative immunofluorescence staining images of E-cadherin (red), α-SMA (green), and DAPI (blue). **(F)** Western blot analysis of fibronectin, collagen I, E-cadherin, and α-SMA in *in vitro* experiment and their relative expression level to GAPDH. Data are presented as the mean ± SD (n = 6). *p < 0.05, **p < 0.01, ***p < 0.001. ns^P > 0.05.

The mRNA and protein levels of fibronectin and collagen I were examined to evaluate ECM deposition (**Figures 2A, B**, respectively). ECM deposition increased in the UUO group but was similarly downregulated in MDSC or GM-CSF groups. This indicated that MDSC transplantation or GM-CSF pretreatment could both inhibit ECM deposition.

We then examined the epithelial marker E-cadherin and the mesenchymal marker α-SMA to evaluate EMT. qPCR and Western blot analysis revealed high E-cadherin expression and low α-SMA expression in the sham group (**Figures 2C, D**, respectively); however, the result was reversed 10 days after UUO, indicating EMT occurrence. However, α-SMA expression decreased and E-cadherin expression increased significantly in the MDSC and GM-CSF groups than those in the UUO group, which were verified using immunofluorescence (**Figure 2E**). These results further confirmed the similar protective role of the two treatments in renal fibrosis.

To further verify the antifibrotic role of BM-induced MDSCs, we conducted an *in vitro* experiment using mTECs. Corresponding to the results of the *in vivo* experiments, Western blot analysis also showed a dose-dependent decrease in ECM and EMT markers after treatment with different MDSC concentrations (**Figure 2F**). Thus, BM-induced MDSCs directly influenced TECs.

### BM-Induced MDSCs and GM-CSF Play Their Renoprotective Role by Inhibiting TGF-β1 Signaling Pathway

**Figure 3:**
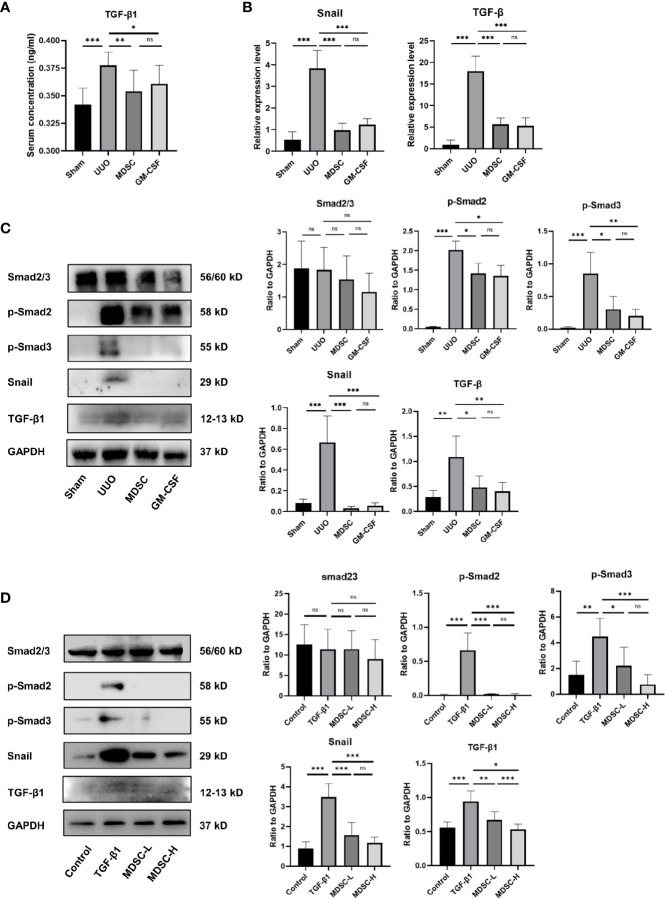
BM-induced MDSCs and GM-CSF play their reno-protective role by inhibiting TGF-β1 signaling pathway. **(A)** Serum concentration of TGF-β1 in all groups. **(B)** Relative mRNA levels of TGF-β1 and Snail in renal tissue. **(C)** Western blot analysis of TGF-β1, Smad2/3, p-Smad2, p-Smad3, and Snail in renal tissues and their relative expression level to GAPDH. **(D)** Western blot analysis of TGF-β1, Smad2/3, p-Smad2, p-Smad3, and Snail in *in vitro* experiment and their relative expression level to GAPDH. Data are expressed as the mean ± SD (n = 6). *p < 0.05, **p < 0.01, ***p < 0.001. ns^P > 0.05.

To explore the mechanism by which MDSCs alleviate renal fibrosis, TGF-β1 expression in serum and kidney tissue were examined using ELISA, qPCR, and Western blotting (**Figures 3A–C**, respectively). TGF-β1 was upregulated significantly in the UUO group, whereas treatments downregulated its expression. We further investigated whether the TGF-β1 signaling pathway was involved in the renoprotective effect of BM-induced MDSCs or GM-CSF. As shown in **Figures 3B, C**, Smad2 and Smad3 phosphorylation was significantly elevated in the UUO group, while both BM-induced MDSCs or GM-CSF significantly ameliorated them. The target gene of the Smad complex, Snail, was also upregulated in the UUO group and downregulated in the two treatment groups. Later, in the *in vitro* experiment, TGF-β1, phosphorylated Smad2 and Smad3, and Snail expression levels in mTEC cells were also upregulated in the TGF-β1 group but downregulated in the MDSC-L group and strongly downregulated in the MDSC-H group (**Figure 3D**). These results suggested that BM-induced MDSCs or GM-CSF alleviates UUO by intervening in the TGF-β1/Smad/Snail signaling pathway. The number of MDSCs may affect their antifibrotic effects.

### Enhanced MDSC Recruitment Is an Important Mechanism for *In Vivo* or *In Vitro* Induced MDSCs to Alleviate Renal Fibrosis

**Figure 4:**
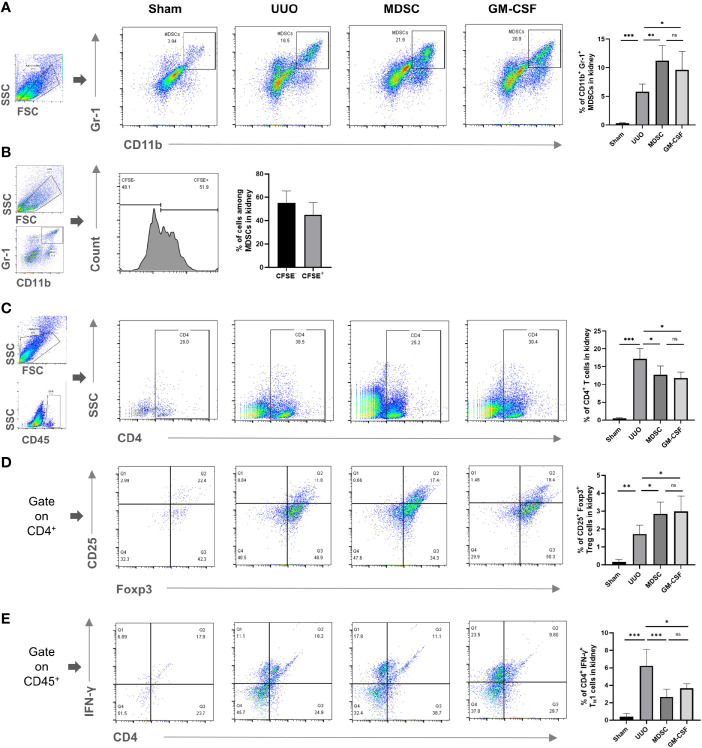
Enhanced MDSC recruitment is an important mechanism for *in vivo* or *in vitro* induced MDSCs to alleviate renal fibrosis. Flow cytometry detected the percentage of **(A)** CD11b^+^ Gr-1^+^ MDSCs in kidney, **(B)** BM-induced MDSCs (CFSE labeled) among all MDSCs in kidney, **(C)** CD4^+^ T cells in kidney, **(D)** CD4^+^CD25^+^Foxp^+^ Tregs in kidney, **(E)** CD4^+^IFNγ^+^ T_H_1 in kidney. Data are expressed as the mean ± SD (n = 6). *p < 0.05, **p < 0.01, ***p < 0.001. ns^P > 0.05.

We then evaluated MDSC accumulation in the kidney after UUO. Compared with that in the sham group, a small number of MDSCs were recruited to the kidneys in the UUO group, while more MDSCs were recruited to the kidneys in both treatment groups (**Figure 4A**). There were no significant differences in the number of MDSCs in the kidneys between the two treatment groups. In addition, among all MDSCs recruited to injury site in the MDSC group, 44.95 ± 0.21% MDSCs were CFSE labeled (incubated with MDSCs before adoptive transference; **Figure 4B**). These results suggest that artificially induced MDSCs (*in vivo* or *in vitro*) have stronger recruitment ability than natural MDSCs, and the recruitment ability of *in vitro*- and *in vivo*-induced MDSCs were comparable.

Observing the MDSC downstream effector cells showed that CD4^+^ T, regulatory T (Treg), and T helper (T_H_) 1 cell recruitment to the kidney increased after UUO (**Figures 4C–E**, respectively). However, the number of T_H_1 cells in the kidney decreased significantly after treatment, while the number of Treg cells increased. These results indicated that MDSCs also alleviate renal fibrosis indirectly by affecting the balance between T_H_1 and Tregs. This effect was related to their elevated recruitment to the injury site.

### Increased CCL5 Expression in *In Vitro-* or *In Vivo-*Induced MDSCs Mediate the Enhanced MDSC Accumulation

**Figure 5:**
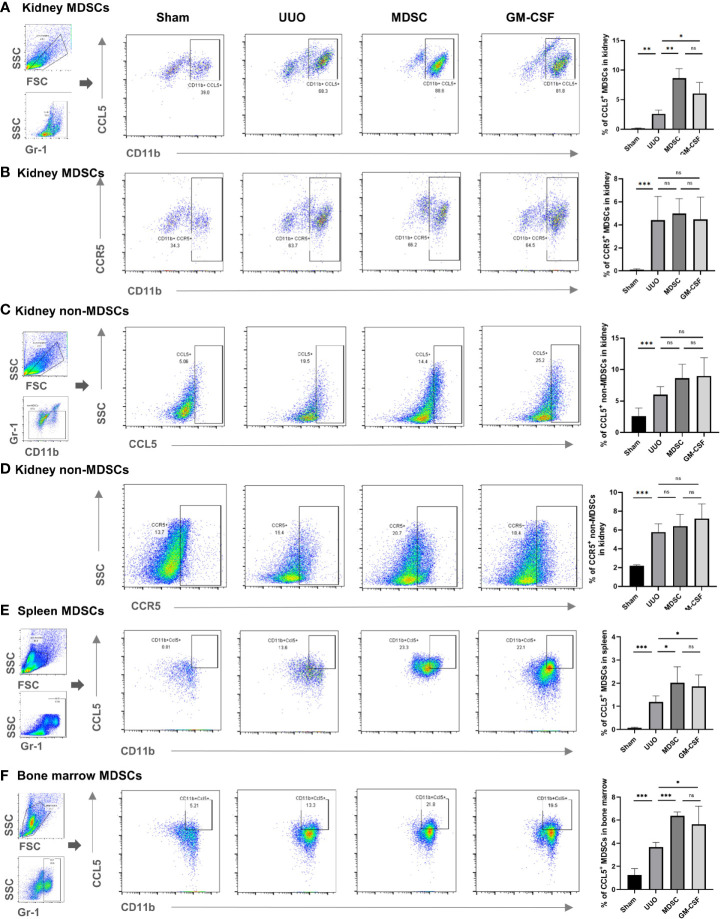
Expression level of CCL5 and CCR5 in MDSCs and non-MDSCs in mice. Flow cytometry detected the percentage of **(A)** CCL5^+^ MDSCs in kidneys, **(B)** CCR5^+^ MDSCs in kidney, **(C)** CCL5^+^ non-MDSCs in kidney, **(D)** CCR5^+^ non-MDSCs in kidney, **(E)** CCL5^+^ MDSCs in spleen, and **(F)** CCL5^+^ MDSCs in bone marrow. Data are expressed as the mean ± SD (n = 6). *p < 0.05, **p < 0.01, ***p < 0.001. ns^P > 0.05.

**Figure 6:**
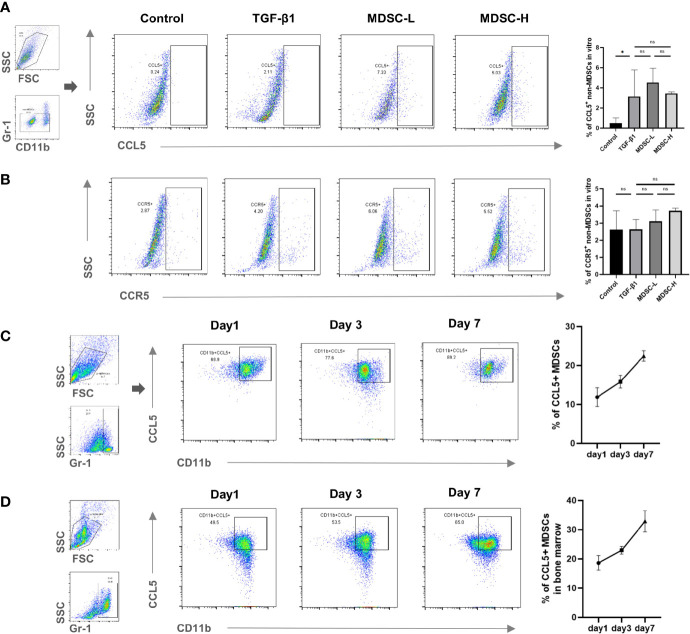
CCL5 expression in *in vitro-* and *in vivo*-induced MDSCs. Flow cytometry detected the percentage of **(A)** CCL5^+^ non-MDSCs in *in vitro* experiment, **(B)** CCR5^+^ non-MDSCs in *in vitro* experiment, **(C)** CCL5^+^ MDSCs in *in vitro*-induced cells, and **(D)** CCL5^+^ MDSCs in bone marrow of mice pretreated with GM-CSF. Data are expressed as the mean ± SD (n = 6). *p < 0.05. ns^P > 0.05.

To explain the mechanism of enhanced recruitment ability of *in vitro*- or *in vivo*-induced MDSCs, the CCL5 and CCR5 expression levels in MDSCs and non-MDSCs of different tissues (kidney, spleen, and BM) were measured. CCL5 expression increased in the MDSCs located in all tissues in the UUO group than that in the sham group and further increased in the two treatment groups (**Figures 5A, E, F**). However, although CCR5 expression also increased in non-MDSCs located in the kidneys after UUO, they did not differ between UUO and the two treatment groups (**Figure 5D**). Moreover, CCR5 expression level in MDSCs and CCL5 expression in in non-MDSCs were also comparable between UUO and the two treatment groups (**Figures 5B, C**, respectively). *In vitro* experiments further confirmed that the CCL5 and CCR5 expression levels in non-MDSCs (mainly mTEC cells) were not altered (**Figures 6A, B**).

Finally, we tracked CCL5 expression in *in vitro*- and *in vivo*-induced MDSCs. *In vitro*-induced MDSCs were harvested and examined 1, 3, and 7 days after induction. *In vivo*-induced MDSCs were separated from the BM of mice 1, 3, and 7 days after GM-CSF treatment. We found that CCL5 increased gradually for 7 days after *in vitro* induction (**Figure 6C**), and the expression level in *in vivo*-induced MDSCs were similar (**Figure 6D**). Thus, *in vitro* and *in vivo* MDSC induction stimulate CCL5 expression. This may also explain the comparable ability of MDSCs to recruit and alleviate renal fibrosis in both regimens.

## Discussion

**Figure 7:**
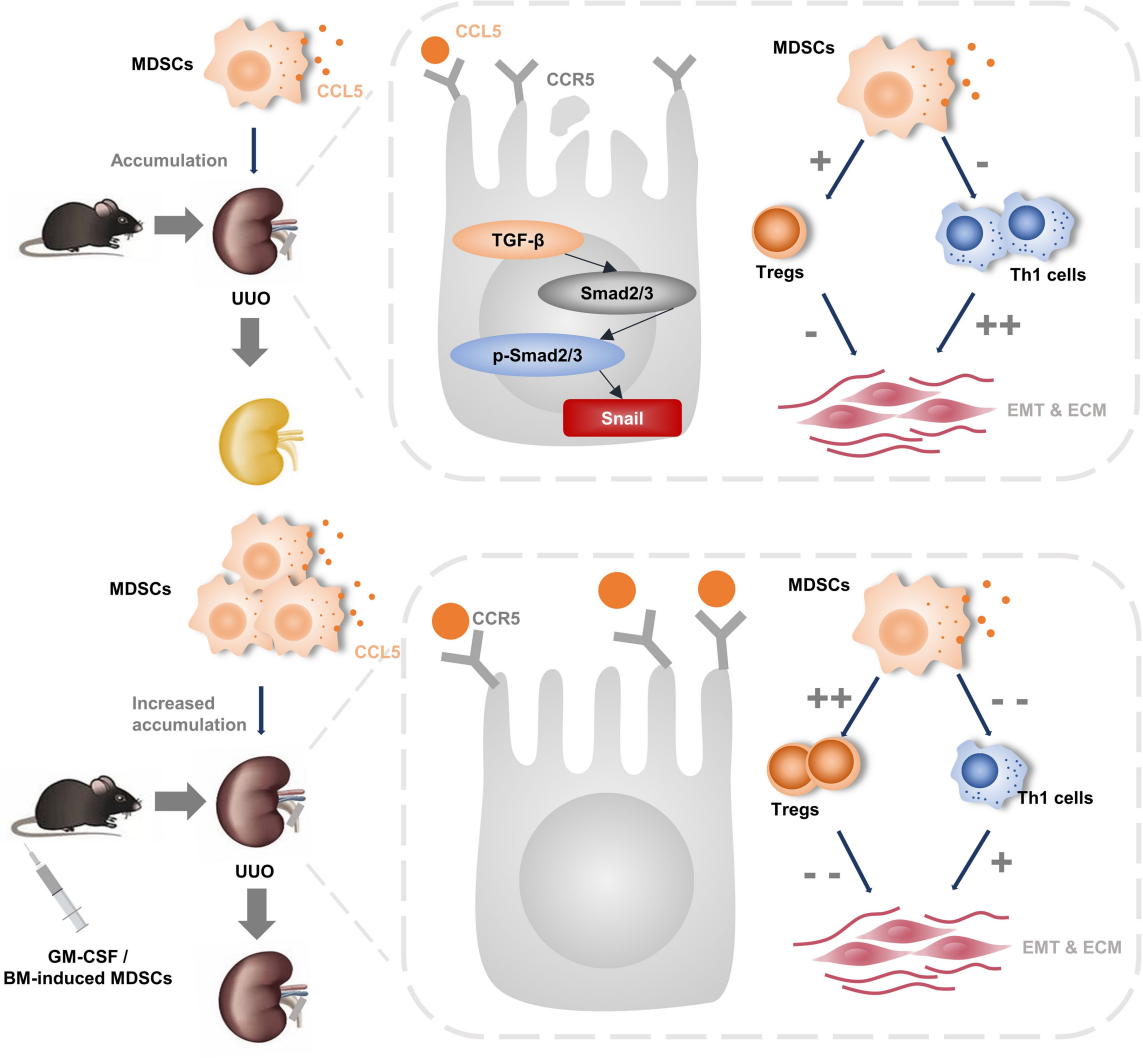
The pictorial summary of induced MDSCs on renal fibrosis. *In vitro-* and *in vivo-*induced MDSCs increased their infiltration to injury by promoting CCL5-CCR5 interaction. In addition, they alleviate renal fibrosis to a similar extend by inhibiting TGF-β1/Smad/Snail signaling pathway and rebalance Th1/Treg.

In this study, we found that BM-induced MDSCs or GM-CSF pretreatment increased MDSC infiltration and significantly ameliorated UUO-induced tubulointerstitial fibrosis. TGF-β1 inhibition and its downstream signaling pathway were involved in this renoprotective process. We further elucidated that induced MDSCs, both *in vitro* and *in vivo*, enhanced CCL5 secretion, which promoted their recruitment to kidneys to play immunosuppressive and anti-fibrotic roles (**Figure 7**).

Overexpression of ECM components, such as fibronectin and collagen I, causes ECM deposition (30), thereby destroying the kidney parenchyma and deteriorating renal function, resulting in renal fibrosis progression. In our study, Masson and Sirius red staining showed increased ECM deposition in UUO group, while adoptive transference of MDSCs or GM-CSF pretreatment attenuated it. Western blotting and qPCR showed that fibronectin and collagen I expression also changed similarly. These results demonstrated that adoptive transference of MDSCs or GM-CSF pretreatment effectively ameliorate ECM deposition and eventually delay renal fibrosis progression.

EMT is a process that depicts the transition from epithelial cells to mesenchymal cells, which contributes a lot to the progression of renal fibrosis. TECs acquiring a mesenchymal phenotype have been deemed as the main source of activated myofibroblasts in the injured kidney (31), and they secrete a wide range of proinflammatory cytokines, which further aggravate renal fibrosis. In our study, EMT activation was also observed through immunofluorescence in the UUO group. Western blot and qPCR also confirmed the reduced E-cadherin and elevated α-SMA expression. However, adoptive transference of MDSCs or GM-CSF pretreatment reversed this trend and caused weight gain in the kidneys. These findings further confirmed the renoprotective role of MDSCs and GM-CSF.

TGF-β1 belongs to the TGF-β superfamily and is considered to be an essential mediator of renal fibrosis (22, 23). It promotes ECM production and EMT (32) by regulating inflammatory cells, including T cells and macrophages (23, 24). The TGF-β1/Smad signaling pathway is one of the canonical fibrogenic growth signaling pathways. Once TGF-β1 binds to its receptor, Smad2 and Smad3 phosphorylation is triggered, and the Smad complex is formed by the combination with Smad4. The Smad complex is then transferred to the nucleus to drive the expression of target genes, including Snail, which also contributes to EMT (25) and fibrosis (26, 27). In this study, we detected significant TGF-β1/Smad/Snail signaling pathway activation in the UUO group, which was inhibited by adoptive transference of MDSCs or GM-CSF pretreatment. These phenomena indicate that MDSCs or GM-CSF play a renoprotective role partly by counteracting the TGF-β1/Smad/Snail signaling pathway.

Immune factors, including immune cells, inflammatory cytokines, chemokine receptors, and their ligands, may contribute to renal fibrosis progression (2–7). Recently, researchers have focused on the effect of MDSCs in renal fibrosis (12, 33). MDSCs develop in various chronic inflammatory conditions, such as infection, tumors, autoimmune diseases, and graft-*versus*-host disease (GVHD). It suppresses T helper (T_H_) cell accumulation and induces Tregs through signal transducer and transcription activator (9). Tregs further inhibit CD4^+^ T_H_ cell proliferation and function by secreting cytokines such as IL-10 and inhibiting IL-2 availability for CD4^+^ T_H_ cells (34, 35). The balance between T_H_1 and Treg cells has drawn great attention in cancer (36), various inflammatory diseases (37, 38), and autoimmunity diseases (39). Treg accumulation and T_H_1 cell downregulation are also necessary for the convalescence of crescentic glomerulonephritis (40). In our study, both *in vitro*- or *in vivo*-induced MDSCs promoted MDSC and Treg infiltration in the kidney and downregulated the total CD4^+^ T and T_H_1 cell infiltration in the kidney. These results suggest that Tregs might partially contribute to MDSC/GM-CSF-induced renal fibrosis remission, which may be mediated by MDSC-induced immunosuppression.

Although natural MDSCs increase their number in inflammation, their immunosuppressive activity might be limited in a highly inflammatory environment due to myeloid differentiation, inflammatory mediator production, and cell-intrinsic inflammasome upregulation (41). Nevertheless, researchers have found that *in vitro*-induced MDSCs after culturing BM cells with G-CSF and GM-CSF obtained a phenotype similar to that of natural MDSCs with a protective effect in various primary target organs and inhibited GVHD-correlated death by 80% (42, 43). This indicated the potent treatment efficiency of adoptive transference of MDSC-enriched populations after *in vitro* expansion. However, the mechanism underlying the maintenance of the immunosuppressive phenotype by *in vitro*-induced MDSCs remains unclear. In our study, approximately half of the MDSCs accumulated to the injury site were BM induced, which further inhibited T_H_1 and promoted Treg expansion. In addition, *in vitro* experiments showed that a high MDSC dose had a strong ability to inhibit ECM and EMT. These results suggest that the number of MDSCs accumulated may have an effect on the maintenance of the immunosuppressive phenotype.

The clinical application of GM-CSF is more convenient than MDSC-adoptive therapy. However, whether they can achieve similar efficacy in renal fibrosis remains unclear. A recent study has reported that enhanced G-CSF and GM-CSF expression promotes MDSC generation and migration (12, 44), indicating that G-CSF and GM-CSF are both efficient for inducing MDSC expansion. However, although G-CSF is more commonly used in clinical settings, GM-CSF is well accepted for *in vitro* MDSC induction because it can induce both MDSC phenotypes (CD11b^+^Ly6G^int^Ly6C^low^ and CD11b^+^Ly6G^int^Ly6C^low^ populations) in a balanced manner. However, their ability to induce MDSCs or immunosuppression *in vivo* remains controversial (45, 46). Thus, we selected GM-CSF for simple and effective MDSC induction. In accordance with our expectations, GM-CSF pretreatment promotes the MDSC infiltration and significantly ameliorates renal fibrosis. The adoptive transference of BM-induced MDSCs showed a similar efficacy. Thus, our results proved the equivalent efficacy of GM-CSF and BM-induced MDSCs and provided an alternative and convenient method for treating renal fibrosis.

The CCL5–CCR5 axis is involved in MDSC recruitment and its immunosuppressive role (19, 47). A previous study has also showed that IL-6 and GM-CSF upregulate CCR5 expression during *in vitro* MDSC induction (18). However, we did not find an increase in CCR5 expression in kidney MDSCs or non-MDSCs or *in vitro* experiments in the treatment groups. In contrast, we found that CCL5 was significantly elevated in MDSCs located in the kidney, spleen, and BM in the two treatment groups. We also found that CCL5 expression gradually increased with time. These results indicated that GM-CSF could increase CCL5 expression in both *in vitro*- or *in vivo*-induced MDSCs. Enhanced CCL5 expression further promotes MDSC recruitment to injuries to exert their immunosuppressive function. A recent case–control study has provided evidence for this hypothesis. Researchers found that decreased serum CCL5 and TGF-β concentrations were highly correlated with reduced blood MDSCs and Tregs in patients with recurrent implantation failure (RIF) (48). Previous research also found that MDSCs from CCL5-knockout mice with breast tumors expressed less NOS_2_ and S100A8/9 and showed an immune-stimulatory phenotype (21). All these studies indicated that CCL5 plays an important role in the immunosuppressive function of MDSCs.

This study had some limitations. Although UUO is a well-accepted renal fibrosis animal model, it rarely causes renal fibrosis or progressive kidney failure in clinical settings. Thus, whether GM-CSF or MDSC treatment could have similar therapeutic effects in other obstruction-induced renal fibrosis diseases remains to be explored. Second, considering the higher metabolic rate in mice than that in humans, the effective GM-CSF dose used for *in vivo* induction is much higher than that for clinical use. Therefore, further studies should determine the optimal GM-CSF dose for inducing human MDSCs. Third, GM-CSF affects several cell types, including macrophages and granulocytes. Future studies should explore its role in influencing renal fibrosis. Finally, CCL5 and CCR5 are not unique ligand receptors, and they are not the only chemokine or receptor expressed in MDSCs. Future studies should focus on ruling out the involvement of other receptors or ligands.

In conclusion, our study demonstrated that *in vitro*- and *in vivo*-induced MDSCs alleviate renal fibrosis similarly, both by promoting the CCL5–CCR5 axis interaction and TGF-β1/Smad/Snail signaling pathway inhibition. Our results indicate an alternative treatment for renal fibrosis.

## Data Availability Statement

The raw data supporting the conclusions of this article will be made available by the authors, without undue reservation.

## Ethics Statement

The animal study was reviewed and approved by Animal Ethical Committee of Zhongshan Hospital, Fudan University.

## Author Contributions

ZL, RR, and JC: conceived the project, designed the project, and approved the final manuscript. YQ, GT, and YC: drafted the manuscript, conducted the experiments, and extracted and analyzed data. JL, FF, YS, and XZ: conducted the experiments and extracted and analyzed data. All authors contributed to the article and approved the submitted version.

## Funding

This work was supported by the National Key R&D Program of China (2018YFA0107501), National Natural Science Foundation of China (81770747, 81970646, and 82070085), construction program of key but weak disciplines of Shanghai Health Commission (2019ZB0105), Natural Science Foundation of Shanghai (20ZR1411100), Program of Shanghai Academic/Technology Research Leader (20XD1421000), Clinical Research Funds of Zhongshan Hospital (2020ZSLC38 and 2020ZSLC27), and Smart Medical Care of Zhongshan Hospital (2020ZHZS01).

## Conflict of Interest

The authors declare that the research was conducted in the absence of any commercial or financial relationships that could be construed as a potential conflict of interest.

## Publisher’s Note

All claims expressed in this article are solely those of the authors and do not necessarily represent those of their affiliated organizations, or those of the publisher, the editors and the reviewers. Any product that may be evaluated in this article, or claim that may be made by its manufacturer, is not guaranteed or endorsed by the publisher.
