# Corrigendum: Myeloid-Derived Suppressor Cells Alleviate Renal Fibrosis Progression *via* Regulation of CCL5-CCR5 Axis

**DOI:** 10.3389/fimmu.2021.804228

**Published:** 2021-11-10

**Authors:** Yue Qiu, Yirui Cao, Guowei Tu, Jiawei Li, Ying Su, Fang Fang, Xuepeng Zhang, Jing Cang, Ruiming Rong, Zhe Luo

**Affiliations:** ^1^ Department of Critical Care Medicine, Zhongshan Hospital, Fudan University, Shanghai, China; ^2^ Department of Anesthesiology, Zhongshan Hospital, Fudan University, Shanghai, China; ^3^ Department of Urology, Zhongshan Hospital, Fudan University, Shanghai, China; ^4^ Shanghai Key Laboratory of Organ Transplantation, Shanghai, China; ^5^ Department of Critical Care Medicine, Xiamen Branch, Zhongshan Hospital, Fudan University, Xiamen, China

**Keywords:** renal fibrosis, CCL5, CCR5, migration, myeloid-derived suppressor cells

In the published article, there was an error regarding the affiliation for author **Jing Cang**. Instead of “**4**”, it should be “**2**”.

The authors apologize for this error and state that this does not change the scientific conclusions of the article in any way. The original article has been updated.

## Publisher’s Note

All claims expressed in this article are solely those of the authors and do not necessarily represent those of their affiliated organizations, or those of the publisher, the editors and the reviewers. Any product that may be evaluated in this article, or claim that may be made by its manufacturer, is not guaranteed or endorsed by the publisher.

